# A Single Wavelength Mid-Infrared Photoacoustic Spectroscopy for Noninvasive Glucose Detection Using Machine Learning

**DOI:** 10.3390/bios12030166

**Published:** 2022-03-07

**Authors:** Abdulrahman Aloraynan, Shazzad Rassel, Chao Xu, Dayan Ban

**Affiliations:** 1 Department of Electrical and Computer Engineering, University of Waterloo, 200 University Ave. W, Waterloo, ON N2L 3G1, Canada; shazzad.rassel@uwaterloo.ca (S.R.); chao.xu.1@uwaterloo.ca (C.X.); 2 Waterloo Institute for Nanotechnology, University of Waterloo, 200 University Ave. W, Waterloo, ON N2L 3G1, Canada; 3 Department of Electrical Engineering, Umm Al-Qura University, Makkah 21955, Saudi Arabia

**Keywords:** noninvasive glucose detection, photoacoustic spectroscopy, mid-infrared spectroscopy, machine learning

## Abstract

According to the International Diabetes Federation, 530 million people worldwide have diabetes, with more than 6.7 million reported deaths in 2021. Monitoring blood glucose levels is essential for individuals with diabetes, and developing noninvasive monitors has been a long-standing aspiration in diabetes management. The ideal method for monitoring diabetes is to obtain the glucose concentration level with a fast, accurate, and pain-free measurement that does not require blood drawing or a surgical operation. Multiple noninvasive glucose detection techniques have been developed, including bio-impedance spectroscopy, electromagnetic sensing, and metabolic heat conformation. Nevertheless, reliability and consistency challenges were reported for these methods due to ambient temperature and environmental condition sensitivity. Among all the noninvasive glucose detection techniques, optical spectroscopy has rapidly advanced. A photoacoustic system has been developed using a single wavelength quantum cascade laser, lasing at a glucose fingerprint of 1080 cm${}^{-1}$ for noninvasive glucose monitoring. The system has been examined using artificial skin phantoms, covering the normal and hyperglycemia blood glucose ranges. The detection sensitivity of the system has been improved to ±25 mg/dL using a single wavelength for the entire range of blood glucose. Machine learning has been employed to detect glucose levels using photoacoustic spectroscopy in skin samples. Ensemble machine learning models have been developed to measure glucose concentration using classification techniques. The model has achieved a 90.4% prediction accuracy with 100% of the predicted data located in zones A and B of Clarke’s error grid analysis. This finding fulfills the US Food and Drug Administration requirements for glucose monitors.

## 1. Introduction

Diabetes mellitus, commonly known as diabetes, is a metabolic disorder that elevates the glucose percentage in the blood, caused by a dysfunction in the production (type-1) or effectiveness (type-2) of insulin in the body. Worldwide, 530 million people have diabetes, causing more than 6.7 million deaths, according to the International Diabetes Federation (IDF) in 2021 [1]. The number of diagnosed diabetics is rapidly and continuously growing, which draws attention to the demand for developing better functional blood glucose monitors. In addition, hypoglycemia is a condition where the blood glucose concentration is dangerously low. Typical blood glucose levels in adults, under various conditions, are shown in Table 1. Both diabetes mellitus and hypoglycemia conditions significantly impact human life and need to be continuously monitored. The current traditional technologies for measuring blood glucose are based on invasive methods. These methods are considered to be painful and inconvenient due to multiple daily blood drawings. Hence, there is demand for the development of new noninvasive technologies that will improve the life quality of those living with diabetes.

The blood glucose concentration can be potentially measured directly from blood, serum, plasma, urine, saliva, and tear liquid, as per [2,3,4,5,6]. Furthermore, it can be directly determined from the interstitial fluid (ISF) [7], located underneath the skin in the epidermis layer. The ISF is a thin layer of bio-fluid located between the cells, composed of water solvent and blood vessels. It contains sugars, fats, amino acids, hormones, coenzymes, white blood cells, and cell waste-products [8]. The glucose diffuses from the blood to the ISF layer within a 5 to 15 min delay period, creating a significant opportunity for the ISF to be a promising target for noninvasive blood glucose monitoring systems [9].

**Figure 1:**
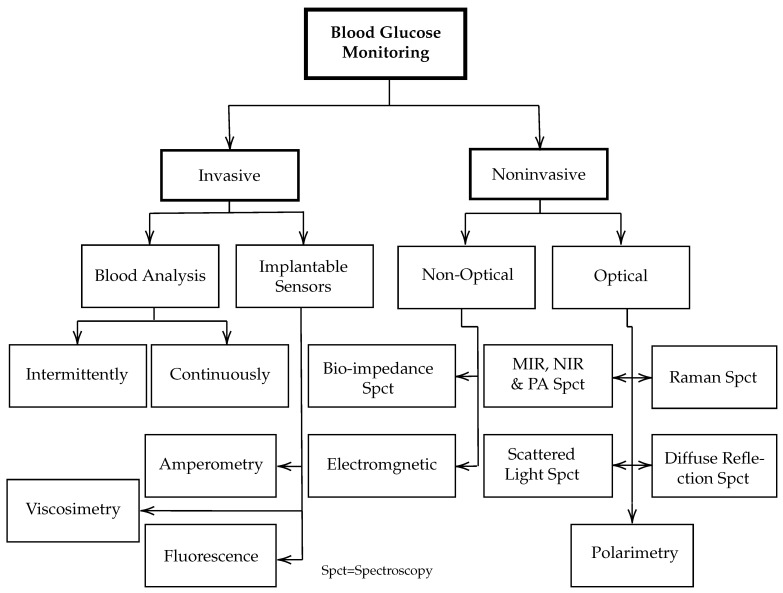
Overview of various techniques and active research areas for in vivo and in vitro glucose monitoring.

Researchers have explored different approaches, including Raman spectroscopy [10,11,12], optical tomography [13,14], and impedance spectroscopy [15,16]. Nevertheless, none of these approaches have yet met the physiological necessity because of their operational instability and low accuracy [17]. Other minimally invasive techniques have been developed. However, they require iterative surgical implantation for the sensors and raise a skin irritation dilemma [18]. The minimally invasive glucose monitoring requires extracting the ISF from the human body without pricking. Figure 1 shows some of the current techniques and active research areas for invasive and noninvasive in vivo glucose detection.

Infrared (IR) spectroscopy, including the NIR and MIR regimes, is being developed as an alternative approach to invasive glucose meters [17]. Both NIR and MIR spectroscopies show strong and broad glucose fingerprint absorption, which draws attention to the implementation of these regions in glucose detection applications. NIR spectroscopy is a cost-effective technique that provides longer light path length in biological samples compared to the MIR. However, the MIR region has distinct glucose fingerprints with less interference with other blood components compared to the NIR region.

The combination of MIR and PA spectroscopy has demonstrated promising potential for substituting the invasive glucose monitoring technology [19,20,21,22]. PA spectroscopy can be employed in the vibration modes of the glucose molecules in the NIR and MIR regions as an alternative approach to compensate for the optical losses in both regions. Specifically, water absorption is much weaker for acoustic signals compared to MIR signals. Quantum cascade lasers (QCLs) in the MIR region have the advantage of generating stronger PA signals and demonstrating stability in the measurements. Therefore, acoustic signals can travel deeper with minimum water scattering and easily reach the ISF in the epidermis layer. The absorption of the acoustic waves increases by raising the glucose concentration because of the vibration mode of the C-O-H bonds of sugar [23]. Other blood components were tested by a PA spectroscopy to characterize their vibration frequencies in order to determine the compatible wavenumbers to be employed for glucose detection, as listed in Table 2 [24].

The combination of PA spectroscopy with MIR spectroscopy for glucose measurements was first investigated in 2005 by Lilienfeld-Toal et al. [20]. Two separate QCLs were used to generate heat pulses in the forearm of a human body. The first laser was used at a glucose absorption peak at 1080 cm${}^{-1}$, while the second one was used to remove any background noise at 1066 cm${}^{-1}$ due to strong water absorption. A sensitive microphone was placed inside an acoustic cell to detect the PA signals from the skin, achieving a correlation factor of 0.61. In 2011, Pleitez et al. [25] published a paper to move progress forward with the use of three QCLs in order to detect the glucose level in the palm at two glucose peaks (1084 and 1054 cm${}^{-1}$) and 1100 cm${}^{-1}$ for the background. A twin Helmholtz gas-cell was used as an acoustic cell with a resonance frequency at 2 kHz. The correlation factor (*R*) was improved to 0.7 compared to their previous experiment [20].

Epidermal skin samples in contact with a glucose solution were studied in vitro with a broadly tunable External cavity (EC) QCL by Kottmann et al. [21]. The tuning range was 1010–1095 cm${}^{-1}$ with an 0.90 cm${}^{-1}$ tuning step and an open-ended PA cell of 78 mm${}^{3}$ volume. A glucose detection limit of 100 mg/dL was obtained with a signal to noise ratio (SNR) of 1 and $R^{2}$ = 0.998 at a glucose peak of 1034 cm${}^{-1}$ and 1080 cm${}^{-1}$. The cell was ventilated by constant N${}_{2}$ gas circulation to overcome humidity and water condensation. However, the glucose detection’s sensitivity is considered to be inadequate compared to the US Food and Drug Administration (FDA) requirement of a ±15 mg/dL accuracy limit for detection. A year later, a flexible, non-toxic silver halide optical fiber was proposed by Kottmann et al. [21] for proper light delivery to different spots on the body. A detection limit of 57 mg/dL and SNR = 1 in an aqueous glucose solution was achieved with $R^{2}$ = 0.993. Three years later, a dual-wavelength aspect was employed by the same research group [26] at 1080 cm${}^{-1}$ for the glucose peak and 1180 cm${}^{-1}$ for the background. The acoustic signals were obtained for in vivo glucose detection from the forearm and fingertip of a healthy, fasting volunteer. The prediction limit was improved to ±30 at a confidence level of 90% for a glucose concentration between 90 and 170 mg/dL. To date, this is the highest glucose prediction sensitivity achieved in PA spectroscopy [11]. Nevertheless, the detection sensitivity is still unsatisfactory for clinically approved glucose monitors. Moreover, using two QCLs, or a tunable EC-QCL, overpriced the system cost. Table 3 summarizes recent progress in PA and MIR combined spectroscopy for glucose detection.

In this paper, a photoacoustic (PA) system has been developed using a single wavelength QCL, lasing at a glucose fingerprint of 1080 cm${}^{-1}$ for noninvasive glucose monitoring. Artificial biomedical skin phantoms with similar properties to human skin have been prepared with different glucose concentrations as test models for the setup. The glucose concentrations in the phantoms cover the range of interest for blood glucose levels in healthy individuals and those living with diabetes. The detection sensitivity of the PA and MIR system has improved to ±25 mg/dL for the glucose range of 75 to 300 mg/dL. An ensemble machine learning model has been developed to detect the glucose concentration of the skin samples using classification techniques. The model has achieved 90.4% prediction accuracy with 100% of the predicted data located in zones A and B of Clarke’s error grid analysis (EGA). This finding fulfills the FDA requirements for glucose monitors.

## 2. Materials and Methods

PA spectroscopy is one of the most promising imaging and detecting technologies to have been well developed over time. The extraordinary sensitivity of PA spectroscopy assists in employing this technique in various fields ranging from biomedical and chemical to biology and physics [29,30,31]. The PA spectroscopy concept relies on generating acoustic waves by an electromagnetic source (particularly modulated light). The radiated electromagnetic waves are absorbed by an object, generating acoustic waves through thermal expansion or pressure. These acoustic waves are distinguishable from one material to another and can be detected by sensitive ultrasonic or piezoelectric sensors. The intensity of the light source plays a critical role in generating acoustic waves. Thus, replacing the regular light source with an intensive light source, such as a QCL, improves the intensity of acoustic signals.

A model has been developed by Rosencwaig and Gersho [19] to study solid samples by PA spectroscopy. In this model, six special cases of the generated PA signals of the sample can be distinguished, based on the ratio of sample length (*l*), thermal diffusion of the sample ($\mu_{s}$), and optical absorption depth ($\mu_{a}$). The PA signal amplitude has an identical dependency on the light intensity and gas coupling properties in all cases. This dependency is defined by a factor (*F*) as follows [24]:

(1) $$F=\frac{\gamma \cdot P_{0}\cdot t\left(\lambda \right)\cdot I_{0}\cdot \mu_{g}}{4\sqrt{2}\cdot l_{g}\cdot T_{0}}$$

where $P_{0}$ is the ambient pressure, t(λ) is the wavelength-dependent fiber transmission, $I_{0}$ is the laser intensity, $\mu_{g}$ is the coupling gas thermal diffusion length, $l_{g}$ is the length of the coupling gas, and $T_{0}$ is the ambient temperature. The gamma factor (γ) is the specific heat ratio at constant pressure and volume ($\gamma =C_{p}/C_{v}$). The thermal diffusing length of the coupling gas, or sample, is defined as follows:

(2) $$\mu_{g,s}={\left(\frac{D_{g,s}}{\pi \cdot f}\right)}^{\frac{1}{2}}$$

where $D_{g},s$ is the gas, or sample, thermal diffusivity and *f* is the modulation frequency of the laser. For biological samples, i.e., human skin, which contain high water content, the penetration depth of the NIR or MIR light is small compared to the sample’s length. In the MIR light, the penetration depth is even smaller due to the stronger water absorption in this region. However, this permeation is adequate for creating informative acoustic signals from the skin, where the glucose molecules are diffused. Therefore, the combination of PA spectroscopy with MIR spectroscopy shows potential for a noninvasive glucose detection system.

The amplitude of the periodical acoustic signal ($A_{PA}$) is directly proportional to the laser intensity ($I_{o}$) and absorption coefficient of the sample (α) as follows:

(3) $$A_{PA}\propto \frac{I_{0}\cdot \alpha }{V_{0}\cdot f^{\frac{3}{2}}},$$

where $V_{0}$ is the volume of the cell and *f* is the modulation frequency. Accordingly, by developing an appropriate design of the PA cell and selection of the modulation frequency, the acoustic signals can be improved, leading to enhanced glucose detection sensitivity. Here, the developed system relies on detecting the deviations of the acoustic signals due to the variations of absorption coefficient in the glucose phantoms. Increasing the glucose concentration in the phantoms heightens the absorption coefficient, thus stimulating the absorbance in the sample to generate higher acoustic signals.

### 2.1. Experimental Setup

**Figure 2:**
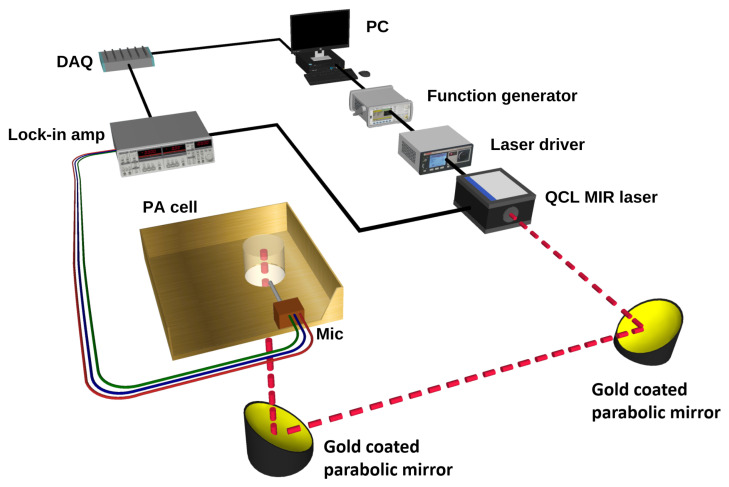
Schematic of the setup used for glucose detection using MIR and PA spectroscopy.

The MIR and PA experimental setup for the noninvasive glucose detection is shown in Figure 2. In this setup, a single wavelength QCL (QD9500CM1, Thorlabs, Newton, NJ, USA) was employed as a light source, lasing at 1080 cm${}^{-1}$ where the glucose has a strong fundamental vibration rotation. The maximum laser power in pulse operating mode was about 60 W with a pulse width of 33 to 100 μs. The laser was operated at 25 °C and had a threshold current of 180 mA. The laser current was frequency-modulated from 10 to 30 kHz with square waves of a duty cycle of 40% by a function generator (Agilent 55321A). The output light of the laser was collimated using an MID lense and placed close to the lasing facet. The beam diameter of the laser was estimated to be less than 2 mm. This laser beam was then reflected upwards to the incident on the PA cell using a gold-coated parabolic mirror with more than 95% reflectivity. A custom-made thermo-electrical cooling (TEC) system was added to the setup to control the temperature during the measurement to provide a sustainable environment. The TEC was controlled by a custom-made proportional-integral-derivative (PID) feedback loop circuit in order to achieve a real-time adjustment [32]. Furthermore, a ventilation system with N${}_{2}$ flow was added to the setup to control the inside humidity of the chamber, preventing moisture from building up on the biological samples.

**Figure 3:**
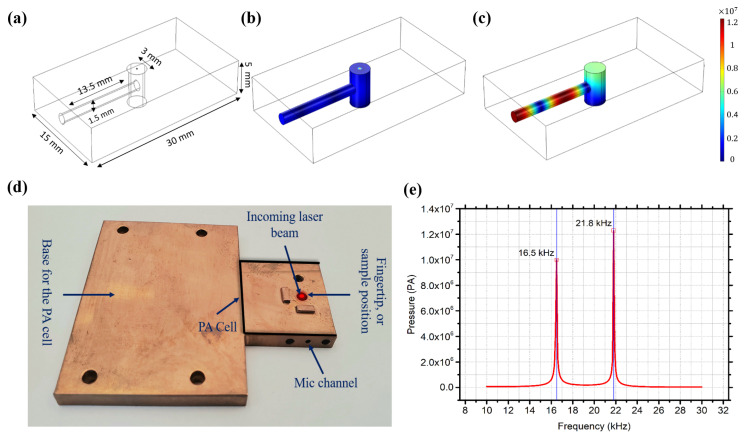
(**a**) PA cell sketch. (**b**) PA off-resonance. (**c**) PA on-resonance. (**d**) Fabricated copper acoustic cell. (**e**) Simulated resonance frequencies of the PA cell.

The PA cell was designed and simulated using COMSOL [33] to collect and amplify the acoustic signals generated in the skin sample or human skin. The PA cell sketch is shown in Figure 3a–c, and the fabricated cell is shown in Figure 3d. The PA cell was made from oxygen-free copper, and the surface was electroplated with gold to prevent oxidation, which may cause a degradation in the thermal conductivity. The length of the laser cavity of the PA cell was 5 mm with a diameter of 3 mm, and the length of the microphone channel was 13.5 mm with a 1.5 mm length diameter. The resonance frequencies of the cell were at 16.50 kHz and 21.80 kHz, as shown in Figure 3e. A slight shift to the resonance frequency is expected while conducting the in vivo and in vitro measurements due to the applied pressure on the cavity. A sensitive analog microphone (SPU0410LR5H-QB, Knowles) was attached to the absorption cell for collecting the acoustic signal from the PA cell. The microphone has a maximum sensitivity between 15 to 30 kHz in order to synchronize with the PA cell resonance frequencies. The PA cell was designed to accommodate both human fingertips and phantom samples to be perpendicularly irradiated by the MIR laser through the PA cavity. Moreover, the PA cell was surrounded by acoustic absorption panels in order to eliminate any environmental background acoustic noises.

### 2.2. Skin Sample Preparation

Human skin consists of complex components that interfere with each other, influencing the PA signals from glucose. The impact of each blood component on glucose was not thoroughly studied in the literature. In biomedical applications, phantoms are widely used as test models to substitute targeted body objects. Here, following the work of Lazebnik [34], artificial skin phantoms were prepared at different glucose concentrations to be used as the test models for a developed system. The skin phantoms can also cooperate in studying the blood components’ interference with glucose in a well-controlled environment by studying the effect of each component individually. This advantage assists in studying the effect of human skin variation and blood components on glucose detection.

The oil-in-gelatin phantoms represent the dielectric properties of various human soft tissues over broadband frequency for biomedical studies purposes. A 200 bloom gelatin derived from calfskin (Sigma-Aldrich, Oakville, ON, Canada) was used as the substantial material for the artificial skin samples. A p-toluic acid (powder) and n-propanol were added to deionized (DI) water and mixed with the gelatin before heating the mixture in a double boiler. After the mixture becomes transparent, the desired ratio of oil is added when the mixture reaches 50 °C. An Ivory ultra liquid detergent surfactant was then added with a formaldehyde solution to provide cross-linking with gelatin. Finally, a D(+)-glucose powder (Sigma-Aldrich) was added to produce glucose concentrations that ranged from 75 to 300 mg/dL with a glucose step of ±25 mg/dL. The mixture was then poured using syringes (to reduce blistering) into specific silicon molds to consolidate for five days. These molds were selected to provide shapes similar to human fingertips (20 mm × 20 mm × 10 mm). Three samples of each glucose concentration were made. Different bakers, syringes, and molds were used for each glucose concentration in the sample preparation procedure. In addition, thinner samples at 0 and 1000 mg/dL were prepared for a compatibility test with the optical properties of human skin. The transmission spectra of the thinner samples were measured by an FT-IR (NICOLET iS50R).

### 2.3. Glucose Measurements

The prepared glucose phantoms, ranging from 75 to 300 mg/dL at ±25 mg/dL glucose differences, were used to investigate the ability of the system for noninvasive glucose detection. The glucose range in the samples covers the scope of interest for blood glucose levels in healthy individuals and those with diabetes. Furthermore, the ±25 mg/dL glucose differences in the phantoms aim to raise the detection sensitivity within FDA specifications [35].

The phantom skin samples were individually placed on the PA cell over the resonator cavity at room temperature. A sensitive pressure transducer (400 FSR, Interlink Electronics, Toronto, ON, Canada) was set beneath the samples to measure the applied pressure and ensure appropriate contact with the cell. Pressure was applied to the samples using a vice that moves in an XYZ direction. The pressure effect on the acoustic signals was investigated before detecting glucose. The appropriate applied pressure was determined by applying various pressure levels to the sample of the highest glucose concentration, which generates the strongest acoustic signal. The pressure level ranged from 0 to 9 N/cm${}^{2}$ in order to examine the pressure effect on the acoustic spectrum of the samples. A consistent pressure level of 6 N/cm${}^{2}$ was eventually applied to all glucose phantoms in the measurements.

The modulated laser beam was focused into the PA cell by a gold-coated parabolic mirror. Each sample was scanned from 10 to 30 kHz with a frequency step of 150 Hz. The absorbed laser pulses generate thermal expansions in the skin samples, which are converted to acoustic waves. These waves are amplified inside the PA cavity and detected by a sensitive microphone (SPU0410LR5H-QB) channeled through the PA cell. A lock-in amplifier (SR830) processed the collected PA signals to increase the SNR with a time constant of 300 ms. The measurements were repeated ten times, and the collected acoustic signals were transmitted to the PC through a data acquisition system for further analysis. The experiment was repeated for three days with new samples following similar procedures. Table 4 shows the summary of the three-day measurements. The in vitro experiment is considered as an initial and essential approach in examining the feasibility of the system for noninvasive glucose detection using a single wavelength MIR laser before implementing and developing the setup for in vivo measurements.

### 2.4. Machine Learning Techniques for Glucose Detection

Despite the recent outstanding development, machine learning (ML) has not been utilized in MIR and PA spectroscopy for noninvasive glucose detection. ML models can assist in improving the detection sensitivity to meet FDA requirements. Furthermore, the employment of ML can help to solve the complexity of detecting glucose in the presence of different blood components or at various environmental conditions. In noninvasive optical spectroscopy, ML models can be developed to distinguish glucose signals despite the variations in human skin properties for in vivo measurements.

Both classification and regression techniques can be employed for noninvasive glucose detection applications. The classification techniques result in discrete outputs labeled by distinct classes, while the regression models extract quantitative information. In other words, the prediction output of the classification models is a discrete glucose value compared to the regression methods that predict continuous glucose levels. Consequently, the regression methods are constrained to correlate the entire range of interest for glucose measurements. This results in associating the hyperglycemia, normal, and hypoglycemia range of blood glucose levels, which is one of the challenges in regression techniques. In contrast, the classification techniques address each discrete value independently, with no influence on other glucose levels. Therefore, reducing the differences in glucose levels between the discrete classes results in high prediction sensitivity.

Different regression models have been employed for glucose detection, such as partial least square (PLS) [26,36], principal component (PC) [28], multiple linear regression (MLR) [37], and artificial neural networks (ANNs) [38,39]. However, these regression models were used only to reduce the correlation coefficient error in associating predicted glucose levels with actual values for the range of interest. In contrast, classification techniques, which have been proposed recently for glucose detection, overcame the challenges in the regression methods [40] based on simulated results. The hidden Markov classification (HMM) model was trained to binary classify the simulated results as normal or abnormal blood glucose levels. A similar approach was followed later, using data obtained from the literature [41], as well as toenail samples [42]. Jernelv et al. later employed convolutional neural networks for in vitro glucose detection measurements obtained from online datasets, including NIR and FTIR measurements [43]. However, no actual experimental measurements were conducted. Liu et al. employed four different regression models, namely forward propagation (FP), radial basis function (RBF), recurrent neural networks (RNNs), and back propagation (BP) to detect glucose in aqueous solutions using PA spectroscopy.

In May 2021, Shokrekhodaei et al. employed both regression and classification models in VIS-NIR transmission spectroscopy for in vitro glucose detection in aqueous solutions [44]. Five different methods were used, namely MLR and feed-forward NN for regression models, while K-nearest neighbor (KNN), decision tree (DT), and support vector machine (SVM) were used as classification models. The study concluded that classification models are more efficient in detecting broad glucose ranges from hypoglycemia to hyperglycemia. The classification-based models outperform regression methods because of their ability to address each range independently.

In the proposed modality, an ensemble classification model was used to investigate the capability of ML for measuring the glucose level in the skin samples using the unprocessed raw data of the acoustic spectrum. After enhancing the system performance, the classification technique was applied to consolidate the power of both the built optical system and ML. The main objective of involving ML is to enhance glucose detection sensitivity in the presence of other blood components.

#### Ensemble Classification Model

**Figure 4:**
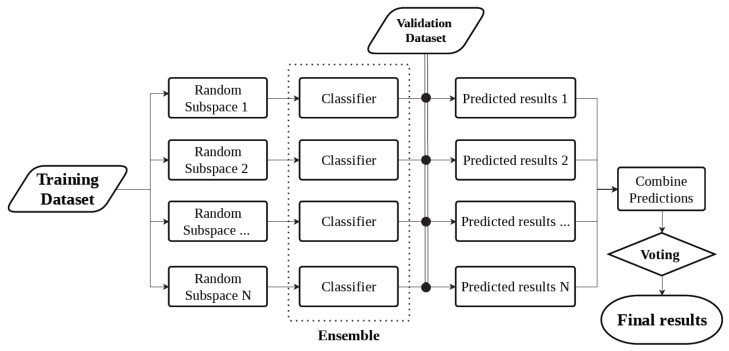
Overview of ensemble machine learning technique using random subspace sampling.

The architecture of the ensemble classification model, using subspace sampling, is presented in Figure 4. Since not all frequencies in the acoustic spectrum provide relevant information for glucose signals, random subspace sampling [45] for the ensemble method was used. The subspace sampling algorithm extracts random features from the spectrum, providing varied outlooks on the data. Thus, individual classifiers are trained using different subspace datasets. The ensemble learning combines several individual models that operate inherently parallel in order to achieve better prediction performance. The ensemble classification learning has shown encouraging results in predictive modeling of type-1 diabetes [46].

In order to generate adequate data for ML, each glucose sample was scanned ten times from 10 to 30 kHz, with a frequency step of 150 Hz. The measurement was then repeated for two more days using different samples, creating 4020 datasets for each glucose concentration, which led to 40,200 datasets for the entire glucose samples, ranging from 75 to 300 mg/dL. Generating a large number of data points assists the training development of ML models, while the arrangement of these data plays a critical role in the efficiency of the models. In ML, each column represents a feature while each row represents a dataset. Therefore, it is essential to ensure that each value in the column is correlated to create one feature for the algorithm. In this work, the data points at every frequency were assigned to one column to create a unit feature for the model with a given class label. In other words, each round of the measurements was converted into a vector before combining them in one matrix. This data arrangement produced 134 features with 30 datasets for each glucose class, as shown in Table 5. The 134 columns represent the frequency range of the measurements from 10 to 30 kHz with a 150 Hz frequency step.

The measured acoustic spectrum for skin phantoms was classified into ten classes based on the glucose concentration of each phantom set. The first six classes cover the glucose level in the normal range (75–200 mg/dL), and the other four classes include the hyperglycemic range (225–300 mg/dL) for fasting and after eating conditions. The data points of the measurements serve as training data for the machine learning classification algorithm, while the glucose class serves as the training data response.

The classification models are trained to predict the class labels using the unprocessed acoustic spectrum of the skin glucose samples in the presence of water and lipids. The aim was to examine the ability of the ML algorithm to classify precisely each glucose concentration without preprocessing to the obtained acoustic signals from the skin samples. The number of learners and the subspace dimension were tuned over the training to maximize the prediction accuracy. The number of learners for the current dataset was tuned between 20 to 50, and 50 to 75 for the subspace dimension. The model was evaluated using the k-fold cross-validation mechanism with 10-fold cross-validation. The dataset is split into ten folds with the same approximate size. One of the nine folds serves as a validation set to evaluate the classifier, while the other nine are used to train the model. This process is repeated until each of the ten folds is employed as a validation set.

In the previous step, the ensemble model was trained with the raw acoustic data to investigate the ability of the optimized system to detect glucose without preprocessing the data. A model to remove the outlier using the moving median was then built to preprocess the acoustic spectrums. The moving median detection method was adopted because of the significant variation in the acoustic signal due to the amplification around the resonance frequency. The asymmetric moving window of the model was 10.2 with a threshold factor of 2.3.

## 3. Results and Discussion

### 3.1. Optical Properties for the Artificial Skin Phantoms

**Figure 5:**
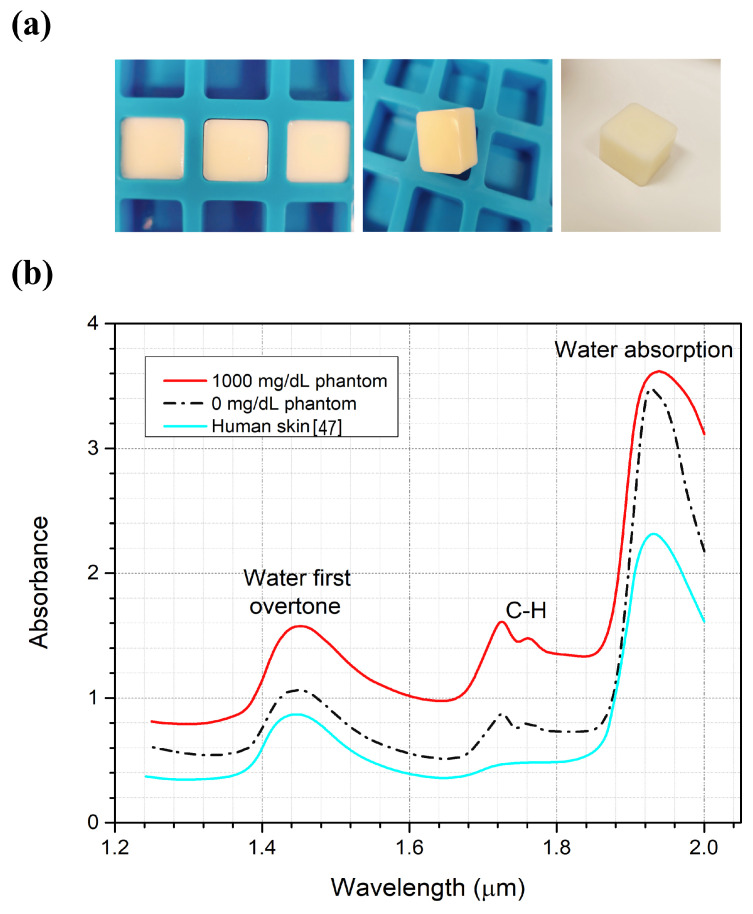
(**a**) Tissue-mimicking phantoms. (**b**) Absorption spectrum for the phantoms compared to the real human skin spectrum.

The prepared tissue-mimicking phantoms, simulating human fingertip size, are shown in Figure 5a. The optical properties of phantoms compared to real human skin are shown in Figure 5b for 0 and 1000 mg/dL glucose concentrations. The dielectric properties of the phantoms were already tested in the work of Lazebnik [34], where the phantoms were prepared for the first time to confirm the similitude of these phantoms to human skin. Here, the optical properties of the tissue-mimicking phantoms were examined and verified to have properties similar to human skin. Furthermore, the sample with a higher glucose concentration shows higher absorbance due to the glucose molecules. The phantoms that were prepared to examine the optical properties were made thinner to allow the IR lights to be transmitted through the samples using the FT-IR. The C-H absorption peak is clearly shown in the fresh samples due to the presence of oil compared to the dry human skin obtained by Delbeck et al. [47]. The oil was added to the sample to examine the glucose detection feasibility in the presence of lipids. This finding enables the employment of these phantoms as test models for biomedical applications that employ optical spectroscopy.

### 3.2. System Optimization

**Figure 6:**
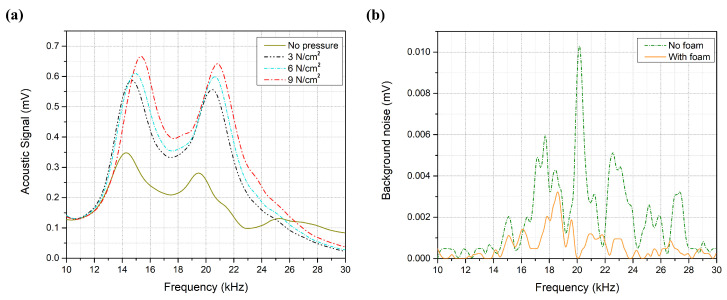
(**a**) Background noise with and without acoustic absorption foam. (**b**) Acoustic spectrum at different pressure levels.

The acoustic absorption panels, which were successfully added to the system, suspend the acoustic background noises with an average of 78% for the entire spectrum, as shown in Figure 6a, which increases the SNR of the system. Furthermore, reducing the background noise around the peaks aids in exposing the glucose buried signals in the acoustic spectrum. The pressure effect is another component that can enhance the SNR of the system. Figure 6b shows the collected acoustic signals at different pressure levels for the 300 mg/dL. The acoustic signal was doubled around the second peak when applying 9 N/cm${}^{2}$ pressure to the sample compared to 0 N/cm${}^{2}$. Increasing the applied pressure level amplifies the acoustic spectrum, which enhances the SNR and the measurement compatibility of the system. However, at the amplification limit of the PA cell, the acoustic signal reaches saturation point, and increasing the applied pressure will not further amplify the collected signals. These findings lead to a significant consequence: the applied pressure has to be lower than the cell’s amplification limit at the highest glucose concentration sample. When the applied pressure exceeds the amplification limit, the acoustic spectrum of the samples will be saturated and will not be further amplified. Thus, the glucose differences among the acoustic spectrum of the samples will be reduced or eliminated. The same concept applies to other parameters that may increase the acoustic signal beyond the amplification limit, such as laser intensity, modulation frequency, and ambient temperature.

### 3.3. Glucose Detection

**Figure 7:**
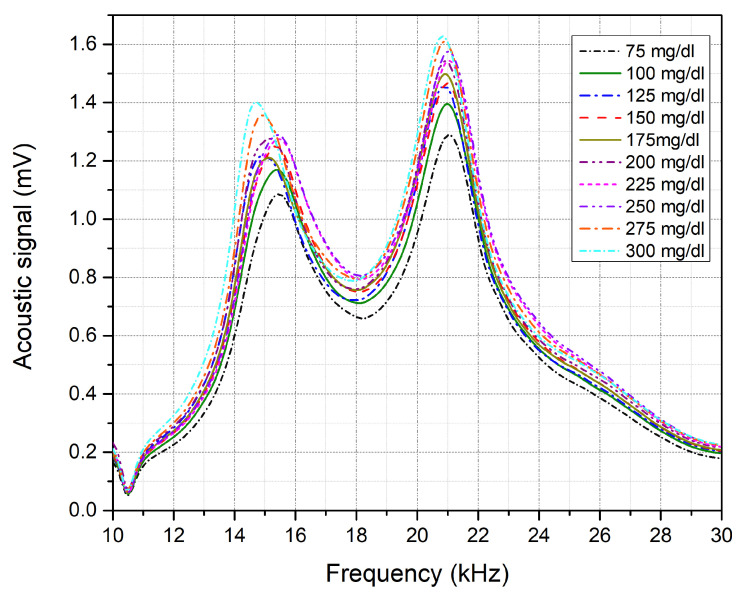
Acoustic spectrum for each glucose skin sample from 75 to 300 mg/dL.

**Figure 8:**
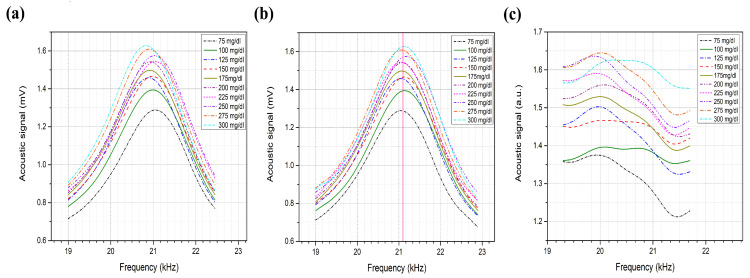
(**a**) Non-rectified acoustic spectrum of the second peak. (**b**) Rectified acoustic spectrum. (**c**) Normalized acoustic spectrum with carbon.

The acoustic spectrum of the glucose phantoms, ranging from 75 to 300 mg/dL, is shown in Figure 7, which shows the average of 10 rounds of measurement for each glucose sample using a single QCL, lasing at 9.25 μm. The glucose difference in the samples was set to ±25 mg/dL, aiming to achieve detection sensitivity that fulfills the FDA requirements. Phantoms with higher glucose concentrations are expected to generate stronger acoustic signals due to the higher absorption of the glucose molecules. The second peak of the collected acoustic spectrum, ranging from 19 to 23 kHz, was found to be sensitive to the glucose levels in the samples. Preliminary results reveal an increment in the acoustic signals along with an increase in the glucose concentrations of the samples. However, frequency shifts were noticed in the spectrum as shown in Figure 8a, which induced glucose detection results. These frequency shifts are attributed to the surface contact of the samples with the PA cell. Accordingly, the acoustic signals must be rectified before obtaining the glucose differences from the signals for the selected frequency range. In the rectification process, all acoustic signals were rectified to have their maximum amplitude at the same frequency. The acoustic spectrums were then normalized with the carbon signal, which was used for the calibration process. The rectification and normalization process is shown in Figure 8b,c.

**Figure 9:**
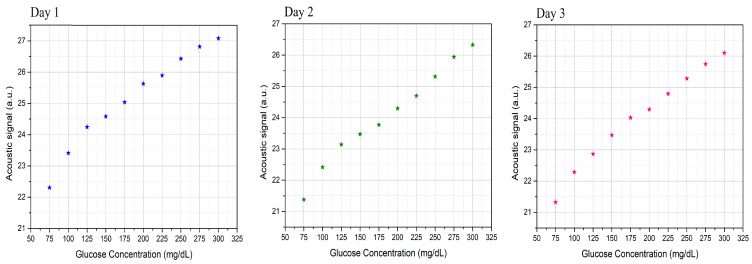
Relationships between the acoustic signals to the corresponding glucose samples for the three-day measurements.

After obtaining the normalized spectrum for the phantoms, the area under the curves was integrated to show the relationship between the acoustic signal to the corresponding glucose sample, as shown in Figure 9. The results, which were conducted without further processing, show that the system was able to distinguish the glucose differences in the skin samples in each of the three days. The presence of glucose molecules in the phantoms increases the absorption of the MIR light, which intensifies the PA of the samples that have higher glucose concentrations. The linear correlation factor of the three-day measurement is R=0.993. The average resolution between the acoustic spectrum of two glucose samples with ±25 mg/dL is 2.3%. These findings raise the detection sensitivity to ±25 mg/dL using a single wavelength QCL for the first time. Moreover, it shows that the system is able to detect glucose for the entire range of interest for blood glucose levels in healthy individuals and those with diabetes.

The system shows sustainability in detecting glucose in the presence of other blood components such as water and lipids. Table 6 shows the standard deviation for the three-day measurements. The deviation in the measurements is associated with a slight degradation in the glucose phantoms over time for the three-day measurements. Moreover, the PA system is sensitive to environmental conditions, causing a variation in the measurements from one day to another. Nevertheless, introducing the temperature and pressure sensors to the setup successfully minimized the deviation in the measurements. Further advancement is required for the in vivo measurements to affirm the system steadiness, in the form of attaching fiber optics and humidity sensors to the setup. Moreover, a comprehensive study is needed to investigate the effect of different skin conditions such as hydration levels and melanin contents on the acoustic signals.

### 3.4. Glucose Detection Using Machine Learning

**Figure 10:**
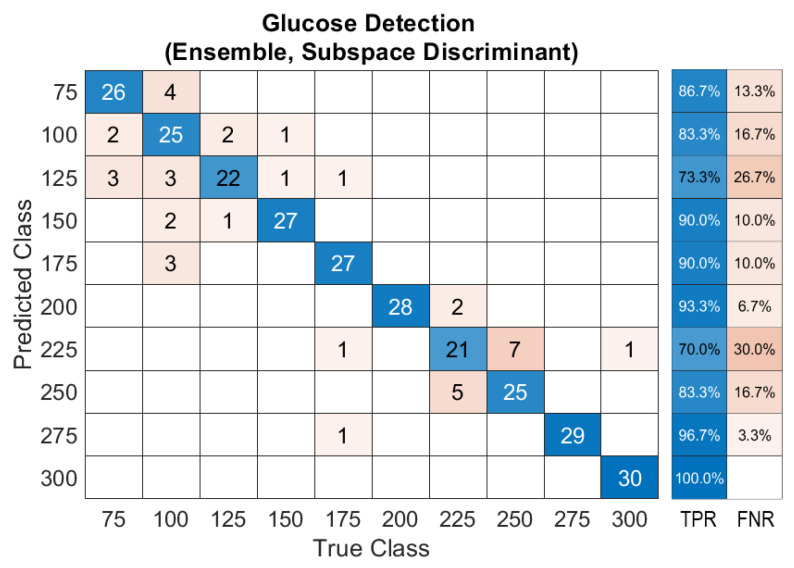
Confusion matrix of the ensemble model for glucose detection.

After demonstrating the system’s feasibility to detect glucose in the skin phantoms using a single light source, ML has been involved in substituting the calibration process for the obtained data. The ensemble classification model was developed using the unprocessed data of the acoustic signals with no rectification or normalization. The ensemble classifier successfully predicted each of the ten classes of glucose concentration from 75 to 300 mg/dL with a prediction accuracy of 86.7%, and an average F1-score of the prediction results of 92.5%. The optimum number of learners was 30, with a subspace dimension of 67. All features were used to train the ensemble model to detect glucose in the samples. The produced confusion matrix of the ensemble classifier is shown in Figure 10. The confusion matrix visualizes the classifier’s performance by representing the true class labels versus the predicted classes. The main diagonal of the confusion matrix demonstrates the number of data samples that are correctly classified. The right-hand side of the confusion matrix shows the percentage of the true-positive rate (TPR) and the false-negative rate (FNR).

**Figure 11:**
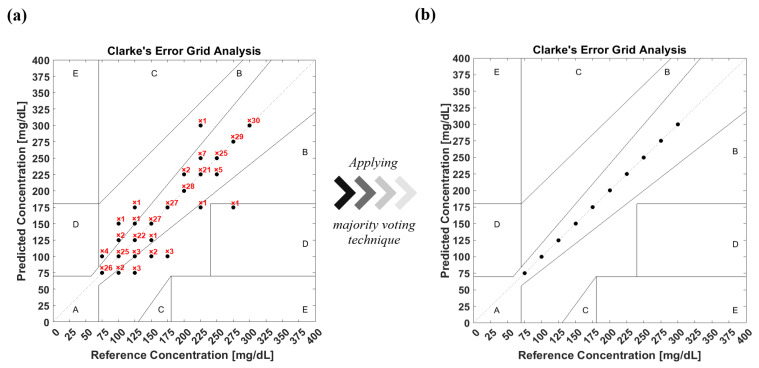
(**a**) Clarke’s EGA of the prediction model of glucose detection before applying the majority voting algorithm. (**b**) Clarke’s EGA of the prediction model after applying the majority voting algorithm.

According to the FDA, 99% of predicted results have to be located within zones A and B in Clarke’s EGA [35], which is used to quantify clinical accuracy for predicted blood glucose measurements to the reference value. In order to evaluate the model’s prediction accuracy, the confusion matrix is converted into Clarke’s EGA, as shown in Figure 11a. The figure shows how many times the classifier predicts the glucose class for each data sample. This results in 93% of the predicted results being located in zone A, while 6.67% and 0.33% are in zones B and D, respectively. A majority voting algorithm was subsequently applied to the prediction data, resulting in reproducing the data in the diagonal line of zone A with 100% accuracy, as shown in Figure 11b. The majority voting algorithm nominates the prediction class based on the number of votes of each class in order to determine the final results.

#### Dataset Preprocessing for ML

**Figure 12:**
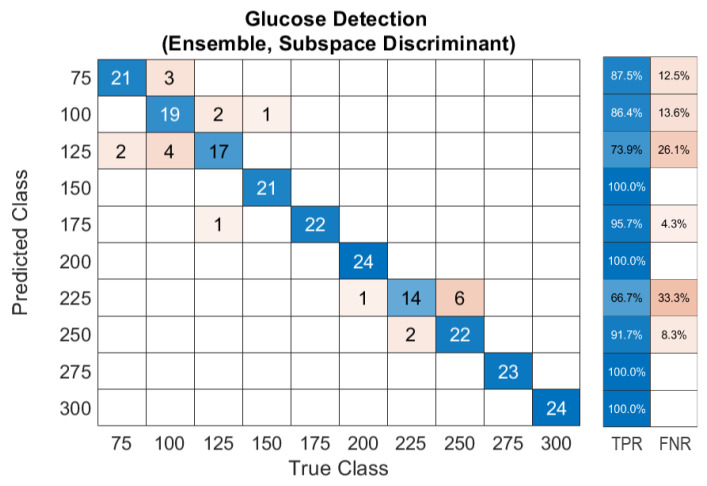
Confusion matrix of the ensemble model trained with preprocessed data of glucose detection.

**Figure 13:**
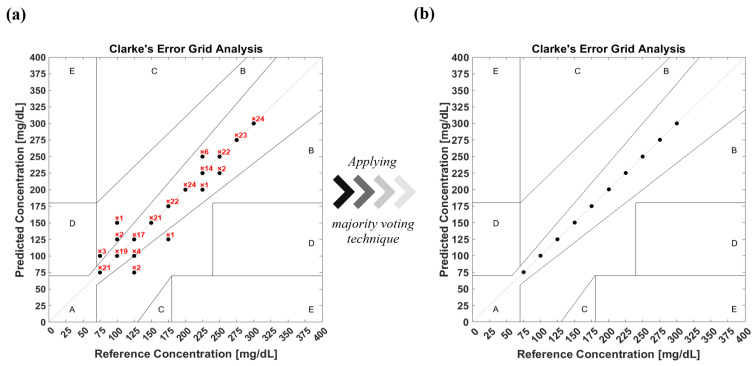
(**a**) Clarke’s EGA of the prediction model with preprocessed data of glucose detection before applying the majority voting algorithm. (**b**) Clarke’s EGA of the prediction model with preprocessed data after applying the majority voting algorithm.

The unprocessed acoustic data has demonstrated sufficient means to train ML models in order to achieve conclusive outcomes that fulfill the FDA guidelines. Nevertheless, the acoustic signals are suspected of environmental conditions, which can introduce unrelated inputs to the system. Therefore, building an algorithm that removes outliers from the data is necessary, particularly when merging in vivo measurements. Removing outliers reduces the validation dataset of the model yet enhances the prediction accuracy. The median moving algorithm improves the ensemble model’s prediction accuracy to 90.4% over the entire glucose range and an average F1-score of 94.5%. The confusion matrix of the new ensemble model with the preprocessed data is shown in Figure 12. The confusion matrix was converted into Clarke’s EGA to quantify clinical accuracy for predicted blood glucose measurements. This results in 96.1% of the predicted results being located in zone A, and with 3.9% in zone B, as shown in Figure 13a. Following similar procedures, the majority voting algorithm was applied to the prediction data to obtain 100% accuracy, as presented in Figure 13b.

## 4. Conclusions

A single wavelength QCL has been employed in a PA and MIR spectroscopy on the glucose fingerprint of 1080 cm${}^{-1}$ for noninvasive glucose monitoring. Artificial biomedical skin phantoms, having similar properties to real human skin, have been prepared to cover the normal and hyperglycemia blood glucose range. The SNR of the system has been effectively enhanced by introducing acoustic absorption panels and pressure sensors. The pressure level applied to the skin phantoms plays a critical role in detecting glucose differences in the PA signals. The PA signals of the highest glucose concentration sample have to be lower than the amplification limit of the PA cell in order to detect the glucose differences. The signal rectification proposed in this work significantly explicates the glucose signal differences in the PA spectrum. The proposed techniques, added to the PA spectroscopy, enable quantifying the glucose level in the samples with the unprocessed acoustic data. The detection sensitivity has been enhanced to ±25 mg/dL using a single wavelength QCL.

An ensemble machine learning model has been developed to classify the glucose concentration in the samples with a 40,200 dataset. The ensemble models trained with an unprocessed and processed dataset achieved 86.7% and 90.4% prediction accuracy, respectively. A majority voting algorithm was applied to both prediction models, resulting in reproducing the data in the diagonal line of zone A of Clarke’s EGA with 100% accuracy. These findings satisfy the FDA standards for glucose monitors.

In vitro measurements conducted in this study are considered to be a significant step in demonstrating the feasibility of the developed PA and MIR system for noninvasive glucose detection. In future works, the glucose sensitivity will be further enhanced before merging into in vivo experiments. The effect of other blood components, such as protein, urea, and cholesterol, on glucose will be investigated using machine learning algorithms. Furthermore, different classification models, such as SVM, NN, and KNN, will be employed and developed for glucose detection.

## Figures and Tables

**Table 1:** Typical blood glucose levels of adult humans.

Condition	Fasting mg/dL	Just Ate mg/dL	3 h after Eating mg/dL
Normal	80–100	170–200	120–140
Pre-diabetic	101–125	190–230	140–160
Diabetic	>= 126	220–300	>200

**Table 2:** Vibrational absorption frequencies for some blood components of the skin [24].

Wavenumber	Component	Intensity
1080 cm${}^{-1}$	β D-glucose absorption	Medium
1080 cm${}^{-1}$	*v*(PO2${}_{2}^{-}$) symmetric	Medium
1077 cm${}^{-1}$	*v*(CC) skeletal trans conformation	Medium
1054 cm${}^{-1}$	α D-glucose absorption	Very weak
1052 cm${}^{-1}$	Albumin absorption	Weak
1047 cm${}^{-1}$	*v*(C–OP)	Weak
1035 cm${}^{-1}$	*v*(CC) skeletal cis conformation	Medium
1034 cm${}^{-1}$	α&β D-glucose absorption	Medium
1020 cm${}^{-1}$	Albumin absorption	Very weak

*v* = stretch.

**Table 3:** Recent progress in PA and MIR combined spectroscopy for glucose detection.

Date	Reference	Source	Wavenumber (cm${}^{-1}$)	Samples	G. conc. (mg/dL)	Correlation or Sensitivity	M.L.	Main Contributions
2005	Toal et al. [20]	QCL	P:1080 Bg:1066	Forearm	0–300	R = 0.61	No	The PA and MIR combination
2012	Kottmann et al. [21]	QCL	P:1034	Epidermal samples	0–2000	±100 mg/dL	No	Using tunable QCLs and N${}_{2}$ ventilation
2012	Pleitez et al. [25]	EC-QCL	P:1054&1084 Bg:1100	Palm	80–260	R = 0.70	R.O.	Selecting three wavelengths
2013	Kottmann et al. [24]	EC-QCL	P:1034	Glucose solution	0–5000	±57 mg/dL	No	Fiber optics for light delivering
2013	Pleitez et al. [27]	EC-QCL	1000–1220	Hypothenar	40–240	-	R.O.	Removing noise by multivariate models
2016	Kottmann et al. [26]	EC-QCL	P:1080 Bg:1180	Fingertip & forearm	90–170	±30 mg/dL	R.O.	Stability improved by increasing pulse rate
2017	Sim et al. [28]	EC-QCL	950–1245	Fingertip & palm	100–250	30%	R.O.	Studying skin effect on measurement

G. conc.: glucose concentration, P: peak, Bg: background, R.O.: regression only.

**Table 4:** Summary of the measurement procedures for glucose detection.

Index	Sample No.	Glucose Level	Round 1	Round 2	…	Round 10	Class Label
Day 1	1st sample	75 mg/dL	10–30 kHz	10–30 kHz	…	10–30 kHz	75
Day 2	2nd sample	75 mg/dL	10–30 kHz	10–30 kHz	…	10–30 kHz	75
Day 3	3rd sample	75 mg/dL	10–30 kHz	10–30 kHz	…	10–30 kHz	75
Day 1	1st sample	100 mg/dL	10–30 kHz	10–30 kHz	…	10–30 kHz	100
.	.	.	.	.	…	.	.
.	.	.	.	.	…	.	.
.	.	.	.	.	…	.	.
Day 3	3rd sample	275 mg/dL	10–30 kHz	10–30 kHz	…	10–30 kHz	275
Day 1	1st sample	300 mg/dL	10–30 kHz	10–30 kHz	…	10–30 kHz	300
Day 2	2nd sample	300 mg/dL	10–30 kHz	10–30 kHz	…	10–30 kHz	300
Day 3	3rd sample	300 mg/dL	10–30 kHz	10–30 kHz	…	10–30 kHz	300

**Table 5:** Dataset arrangement of the glucose acoustic spectrum for ML training purposes.

Index	10 kHz	10.15 kHz	10.30 kHz	…	20.05 kHz	2.20 kHz	…	30 kHz	Class Label
Day 1	round 1	round 1	round 1	…	round 1	round 1	…	round 1	75 mg/dL
	.	.	.	…	.	.	…	.	.
	.	.	.	…	.	.	…	.	.
	round 10	round 10	round 10	…	round 10	round 10	…	round 10	75 mg/dL
Day 1	round 1	round 1	round 1	…	round 1	round 1	…	round 1	100 mg/dL
	.	.	.	…	.	.	…	.	.
	.	.	.	…	.	.	…	.	.
	round 10	round 10	round 10	…	round 10	round 10	…	round 10	100 mg/dL
.	.	.	.	…	.	.	…	.	.
.	.	.	.	…	.	.	…	.	.
.	.	.	.	…	.	.	…	.	.
Day 2	round 1	round 1	round 1	…	round 1	round 1	…	round 1	75 mg/dL
	.	.	.	…	.	.	…	.	.
	.	.	.	…	.	.	…	.	.
	round 10	round 10	round 10	…	round 10	round 10	…	round 10	75 mg/dL
.	.	.	.	…	.	.	…	.	.
.	.	.	.	…	.	.	…	.	.
.	.	.	.	…	.	.	…	.	.
Day 3	round 1	round 1	round 1	…	round 1	round 1	…	round 1	300 mg/dL
	.	.	.	…	.	.	…	.	.
	.	.	.	…	.	.	…	.	.
	round 10	round 10	round 10	…	round 10	round 10	…	round 10	300 mg/dL

**Table 6:** Standard deviation of the three-day measurements using different skin samples.

Glucose Concentration (mg/dL)	75	100	125	150	175	200	225	250	275	300
Standard Deviation	$2.55\times 10^{-2}$	$2.71\times 10^{-2}$	$3.10\times 10^{-2}$	$2.69\times 10^{-2}$	$2.76\times 10^{-2}$	$3.11\times 10^{-2}$	$2.64\times 10^{-2}$	$2.54\times 10^{-2}$	$2.17\times 10^{-2}$	$1.94\times 10^{-2}$
